# The Risk of Misdiagnosing the Primary Site Responsible for Bone Metastases in Patients With Chronic Lymphocytic Leukemia and a Second Primary Carcinoma

**DOI:** 10.14740/wjon873e

**Published:** 2015-04-12

**Authors:** Georges Hatoum, Cyrus Meshkin, Sufana Alkhunaizi, Richard Levene, Julie Formoso-Onofrio

**Affiliations:** a Comprehensive Cancer Center JFK Medical Center, Atlantis, FL 33462, USA; b Hospice & Palliative Medicine Fellowship Program, University of Miami Miller School of Medicine Palm Beach Regional Campus, Atlantis, FL 33462, USA; c Hospice & Palliative Medicine Fellowship Program, Hospice of Palm Beach County, University of Miami Miller School of Medicine Palm Beach Regional Campus, Atlantis, FL 33462, USA

**Keywords:** Chronic lymphocytic leukemia, Renal cell carcinoma, Bone metastasis, Excisional biopsy, Corpectomy

## Abstract

Chronic lymphocytic leukemia (CLL) is a common malignancy which may coexist with other primary cancers. CLL is rarely the cause of solitary bone lesions; such lesions in the context of CLL are believed to result from either Richter’s transformation or metastasis from another primary malignancy. Renal cell carcinoma (RCC), on the other hand, is a malignancy which frequently metastasizes to bone and may cause an osteolytic solitary bone lesion. The origin of a solitary bone lesion in a patient with multiple potential primary malignancies has prognostic implications and affects treatment protocol, and as such must be diagnosed accurately. We describe a patient with CLL and a history of RCC who is found to have an incidental solitary bone lesion of the T11 vertebra. After two separate CT-guided biopsies revealed various lymphoid cell predominance and no evidence of RCC, treatment with low dose external beam radiation therapy (EBRT) was employed. Post-therapy MRI showed further propagation of the lesion. Surgical corpectomy was subsequently performed and postoperative pathology of the lesion was consistent with RCC. The patient was treated with bisphosphonates and a higher dose of EBRT. Our case illustrates the importance of surgical excisional biopsy for accurately diagnosing the primary source metastatic to the bone in a patient with CLL and another potential primary cancer.

## Introduction

Chronic lymphocytic leukemia (CLL) is the most common subtype of leukemia, accounting for approximately 38% of all leukemia diagnoses. According to the American Cancer Society, an estimated 18,500 new CLL diagnoses will be made in 2013 [1]. Diagnosis of a second malignancy with a prior diagnosis of CLL is common. This increased susceptibility is theorized to be secondary to innate defects in immunity as well as immune system depletion from treatment [2]. However, metastasis to bone with isolated CLL is rare; only a few case reports have been published detailing such cases.

Renal cell carcinoma (RCC) comprises 2-3% of all malignancies, and approximately one-third of patients have metastatic disease at time of diagnosis [3]. RCC has a propensity for metastasizing to bone, and may appear on imaging as an osteolytic bone lesion. Patients with solitary bone metastasis from RCC have a better prognosis than those with multiple metastases; their 5-year survival rate is reportedly 35-60% [4]. As compared to other tumors which commonly metastasize to bone, metastatic RCC has a better prognosis than metastatic lung cancer, but worse prognosis than metastatic breast or prostate cancer. This is owing to the relative radio-resistance of most RCCs.

Patients presenting with solitary bone metastasis in the presence of CLL warrant a meticulous workup including a biopsy to precisely identify the responsible primary source. CLL is rarely the cause of metastatic bone lesions; such lesions in the context of CLL are believed to result from either Richter’s transformation or metastasis from another primary malignancy [5]. Furthermore, Richter’s transformation represents a change in the nature of the malignancy; it is characterized by the new onset of high-grade non-Hodgkin’s lymphoma in a patient with CLL [6]. Therefore, in the case of a patient with CLL and another potential primary malignancy who presents with a solitary bone lesion, CLL is unlikely to have caused the lesion.

A CT-guided bone biopsy for diagnosis of vertebral metastases carries a significant sampling error; this sampling error has been documented in the workup of many types of malignant pathology. One 2008 study of 430 CT-guided biopsies showed a 93.3% accuracy, with the highest false negative rate occurring in biopsies of either cervical or non-malignant pathologies [7]. The authors maintained that CT-guided biopsy should be considered the gold standard for biopsies of the spine. However, in the face of high clinical suspicion for radio-resistant vertebral metastases and the possibility of cure with surgical intervention, an excisional biopsy to properly determine the primary source of bone metastases could be of great value.

## Case Report

A 61-year-old white male with a history of CLL diagnosed in 1999 on active surveillance, and prior stage III RCC diagnosed in 2008, presents for ongoing disease management without major complaints. The patient had been compliant with follow-up since treatment and had not required further intervention.

A routine chest computed tomography (CT) scan of the chest revealed a 1.5 × 1.8 cm hypodense lesion involving left anterior aspect of the T11 vertebral body. Subsequent magnetic resonance imaging (MRI) of the thoracic spine confirmed the presence of a hypointense lesion involving T11 which measured 2.8 cm in diameter with no epidural component or spinal cord compression. A whole body bone scan demonstrated a focal uptake in the T11 vertebral body and another focal uptake in the eighth right rib which was attributed to trauma.

A CT-guided biopsy of the T11 vertebral body revealed infiltrates of small lymphocytic cells with a predominant monoclonal B cell population and no evidence of RCC. A repeat spinal biopsy revealed small lymphocytic lymphoma with no evidence of RCC. Accordingly, the patient received palliative external beam radiation therapy (EBRT) to a dose of 400 cGy in 2 fractions targeting the T10, T11, and T12 vertebral bodies.

Despite EBRT, a post-treatment MRI revealed an enlargement of the T11 lesion with focal enhancement and further extension of the tumor across the midline. Given these findings, the patient was advised to be evaluated by the orthopedic oncology service at Mass General Hospital, where he underwent a T11 corpectomy.

**Figure 1:**
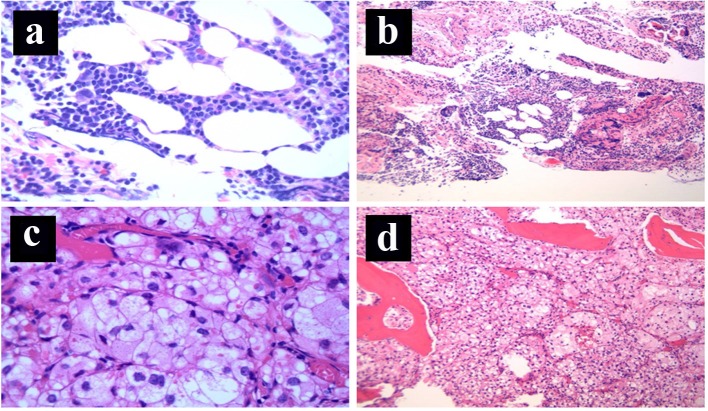
(a) Bone marrow: normal hematopoietic elements (H&E stain, × 100 magnification). (b) Bone marrow: normal hematopoietic elements (H&E stain, × 40 magnification). (c) Bone marrow: replacement of the normal hematopoietic elements by nests of cohesive epithelial cells with prominent clear cytoplasm surrounded by a capillary network, consistent with metastatic clear cell renal cell carcinoma (H&E stain, × 400 magnification). (d) Bone marrow: diffuse replacement of the normal hematopoietic elements by metastatic clear cell renal cell carcinoma (nests of cohesive epithelial cells with prominent clear cytoplasm containing a rich capillary network, H&E stain, × 40 magnification).

Final postoperative pathology was consistent with primary metastatic RCC to the T11 vertebral body with microscopic positive surgical margins of resection. The histopathology is shown in Figure 1c, d with normal hematopoietic elements demonstrated in Figure 1a, b. In accordance with the new diagnosis of metastatic RCC, the patient received a second course of palliative radiotherapy to the surgical bed to a dose of 36 Gy in 2 Gy per fraction, along with biphosphonates.

## Discussion

Patients with CLL and history of another primary malignancy who present with bone metastases warrant a careful initial evaluation. A tissue biopsy is indicated when there is concern for a metastasis in a patient without prior metastasis or when clinical evidence is inconsistent with a diagnosis of bone metastasis [8]. The presence of a solitary bone lesion in this patient should immediately raise concern that the primary source of metastasis is RCC, another primary malignancy, or possibly a manifestation of a Richter’s transformation [6]. Other primary sites with high propensity for bone metastases in males include prostate, lung, and thyroid [9]. Despite the two prior CT-guided needle biopsies which failed to show evidence of RCC in our case, an argument may be made for an excisional biopsy given the very low probability of CLL causing a solitary bone lesion and the relatively high probability of RCC causing such a lesion.

The radiographic appearance of metastatic disease should further guide the clinician in identifying the primary source. Bone lesions in CLL are rare and usually consist of areas of diffuse osteopenia [10], while localized lytic bone metastases are generally secondary to renal, pulmonary or thyroid carcinomas [11]. Therefore, the radiographic studies performed in this case helped to further support the case for RCC as the cause of the metastasis and for excisional biopsy as the next reasonable diagnostic step.

Radiation therapy for bone metastases is largely palliative in nature, but is effective in treating pain from vertebral metastases in over 80% of patients [12]. A select number of patients may require special consideration of aggressive therapy with a curative intent. Given adequate resection with clear microscopic margins, radiation therapy may be avoided entirely. RCC in particular deserves careful consideration of surgery with curative intent owing to its 35-50% 5-year survival and relative radio-resistance [3]. Given this information, our case illustrates a patient who seems to have been a prime candidate for surgical therapy. EBRT was added to his regimen after surgery only due to the positive postoperative surgical margins.

Prior to surgical biopsy of the lesion, low-dose EBRT was administered. This was due to the two sets of biopsy results which were suggestive of CLL. Continued propagation of the lesion post-treatment prompted concern that CLL was an incorrect diagnosis, at which point the patient was advised to obtain a surgical consult for excisional biopsy and surgical removal. Another course of adjuvant palliative radiation therapy to a dose of 36 Gy in 2 Gy per fraction was given to the thoracic spine from T10 to T12. The extended borders for radiation were justified by the positive surgical margins and known radio-resistance of RCC [13].

### Conclusion

We presented the case of a patient with CLL and a history of RCC who was found to have a vertebral metastasis of unclear origin. In this patient, proper treatment required prompt identification of the origin of the metastatic disease. When presented with a presumed vertebral metastasis, early diagnosis and immediate action with correct treatment may reduce morbidity. Our case illustrates the importance of recognizing the limited sensitivity of CT-guided needle biopsy as well as the greater sensitivity of surgical bone excision. In the case of a single vertebral metastasis with typical radiographic appearance and high suspicion of sampling error, surgical bone excision may give a more accurate diagnosis of the underlying disease.
